# The Complete Mitochondrial Genome of Box Tree Moth *Cydalima perspectalis* and Insights into Phylogenetics in Pyraloidea

**DOI:** 10.3390/ani13061045

**Published:** 2023-03-14

**Authors:** Yichang Gao, Jie Zhang, Qinghao Wang, Qiuning Liu, Boping Tang

**Affiliations:** 1 School of Pharmacy, Nanjing University of Chinese Medicine, Nanjing 210023, China; 2 Jiangsu Key Laboratory for Bioresources of Saline Soils, Jiangsu Synthetic Innovation Centre for Coastal Bio-Agriculture, Jiangsu Provincial Key Laboratory of Coastal Wetland Bioresources and Environmental Protection, School of Wetlands, Yancheng Teachers University, Yancheng 224007, China

**Keywords:** Pyraloidea, *Cydalima perspectalis*, mitochondrial genome, phylogenetic analysis

## Abstract

**Simple Summary:**

The mitochondrial genome (mitogenome) has been extensively employed in the investigation of phylogenetic relationships at different taxonomic levels. The mitochondrial genomes of insects are important for understanding their evolution and relationships. Herein, the entire mitogenome of *Cydalima perspectalis* was sequenced and characterized. Comparative mitogenomics and phylogenetic relationships were performed within the Pyraloidea. Our comparative studies show that mitochondrial genomes are a useful tool for phylogenetic studies at the level of the subfamilies in the Pyraloidea.

**Abstract:**

To resolve and reconstruct phylogenetic relationships within Pyraloidea based on molecular data, the mitochondrial genome (mitogenome) was widely applied to understand phylogenetic relations at different taxonomic levels. In this research, a complete mitogenome of *Cydalima perspectalis* was recorded, and the phylogenetic position of *C. perspectalis* was inferred based on the sequence in combination with other available sequence data. According to the research, the circular mitochondrial genome is 15,180 bp in length. It contains 22 transfer RNA genes (tRNAs), two ribosomal RNA genes (rRNAs), 13 typical protein-coding genes (PCGs), and a non-coding control region. The arrangement of a gene of the *C. perspectalis* mitogenome is not the same as the putative ancestral arthropod mitogenome. All of the PCGs are initiated by ATN codons, except for the cytochrome c oxidase subunit 1 (*cox1*) gene, which is undertaken by CGA. Five genes have incomplete stop codons that contain only ‘T’. All tRNA genes display a typical clover–leaf structure of mitochondrial tRNA, except for *trnS1* (AGN). The control region contained an ‘ATAGG(A)’-like motif followed by a poly-T stretch. Based on the mitochondrial data, phylogenetic analysis within Pyraloidea was carried out using Bayesian inference (BI) and maximum likelihood (ML) analyses. Phylogenetic analysis showed that *C. perspectalis* is more closely related to *Pygospila tyres* within Spilomelinae than those of Crambidae and Pyraloidea.

## 1. Introduction

Lepidoptera, with more than 157,000 known species and 137 families among 43 superfamilies, is the world’s third most significant order after Diptera and Coleoptera [1]. One of several Lepidoptera superfamilies, Pyraloidea, includes the Pyralidae and Crambidae families. To date, over 15,500 different species of Pyraloidea have been identified around the world [2]. Pyraloidea insects contain a large number of economically significant pests that affect forests, agriculture, stored goods, and ornamental plants, and they have been used as model insects to research biodiversity, community ecology, management, behavioral ecology, genetics, and the evolution of pheromone communication networks [3,4,5,6,7]. The box tree moth *C. perspectalis* (Walker 1859) (Lepidoptera: Crambidae) is native to East Asia and invasive in Europe; however, it is currently a completely unique species to Middle and South Europe [8]. However, the Pyraloidea classification has not yet reached a satisfactory or stable state. The phylogenetic hypothesis for the higher-level taxa of Pyraloidea was demonstrated through molecular data, which are new and derived from mitochondrial genomes.

Mitochondrial genomes are considered robust phylogenetic relationship markers due to maternal inheritance [9], infrequent recombination [10], a relatively high rate of evolution, and immobile gene components [11]. The insect mitochondrial genomes are often rounded molecules of around 15–16 kb, which include two ribosomal RNA (*rrnL* and *rrnS*) genes, 22 tRNA genes, 37 genes, and 13 PCGs. A non-coding element containing initiation sites for replication and transcription is called the A + T rich region (CR) [12,13]. With the great developments in PCR techniques and high-throughput sequencing, many animal group’s complete mitogenomic data including insects are easier to obtain and have been widely applied in the research of phylogenetics, molecular evolution, evolutionary and comparative genomics, and population genetics [14,15,16].

In this study, we presented a complete mitogenome sequence of *C. perspectalis* and compared its structures with some of the determined Pyraloidea species. Meanwhile, the gene sequence data were incorporated from other available Pyraloidea species listed in GenBank. We also reconstructed phylogenetic trees from PCG sequences to analyze the evolutionary relationships in Pyraloidea insects.

## 2. Materials and Methods

### 2.1. Ethics Standards

The Committee of the Yancheng Teachers University and Nanjing University of Chinese Medicine approved the animal protocols, and all experiments were performed under the applicable standards, with access no. YCTU-2020007 and SP-2020003, respectively.

### 2.2. Sample Collection and DNA Extraction

The moths of *C. perspectalis* were gathered in Yancheng, Jiangsu Province, China. The specimens were stored in 100% ethanol at −20 °C until DNA extraction. The total genomic DNA was extracted from the legs of moths using the Ezup Column Animal Genomic DNA Purification Kit (SangonBiotech, Shanghai, China) in accordance with the manufacturer’s protocol. 

### 2.3. Mitogenome Sequencing

Universal primer sets for mitogenomic sequences from other Lepidopteran insects were designed to amplify the *C. perspectalis* mitogenome [17,18,19,20]. PCR was conducted in the following series: 3 min at 94 °C, followed by 35 cycles of 30 s at 94 °C, 1–3 min at 50–62 °C, and 10 min at 72 °C. All amplifications were conducted in 50 μL reaction volumes using the Mastercycler gradient and Eppendorf Mastercycler. The PCR products were separated by agarose gel electrophoresis (1% *w*/*v*) and then purified using a DNA Gel Extraction Kit (Vazyme, Nanjing, China). The refined PCR products were ligated into T-vector (SangonBiotech, Shanghai, China) and sequenced at least three times.

### 2.4. Gene Annotation and Sequence Assembly

Sequence annotation was applied by NCBI Internet BLAST function for the searching and packaging of MITOS (http://mitos2.bioinf.uni-leipzig.de/index.py (accessed on 10 January 2023)). Alignments of *C. perspectalis* PCGs and different Pyraloidea mitogenomes were applied by MAFFT^17^. The following rules were calculated using composition skewness: GC-skew = [G − C]/[G + C] and AT-skew = [A − T]/[A + T]. Nucleotide composition statistics and codon usage were computed using PhyloSuite [21]. 

### 2.5. Phylogenetic Analysis

GenBank provides the Pyraloidea species used for mitogenomic phylogeny to determine the phylogenetic relationships among Pyraloidea insects based on nucleotide alignments (Table 1). *Spodoptera litura* was used as an outgroup. Using default concatenation and settings, nucleotide sequences were aligned for each of the 13 mitochondrial PCGs. MrBayes v 3.2.2 [22] and IQ-Tree [23] performed phylogenetic analyses using the maximum likelihood (ML) and Bayesian inference (BI), respectively. Each of the PCGs was individually aligned using MAFFT [24]. Gblocks were applied to ensure protected areas and eliminate undependably aligned sequences in the datasets [25]. For ML and BI analyses, GTR + I + G was the suitable model for nucleotide sequences by MrModeltest 2.3 on Akaike’s information criterion (AIC) [26]. Bayesian analysis was conducted under the following circumstances: 10,000,000 generations, four chains, and a burn-in step for the first 5000 generations, 100 sample frequency. We evaluated the reliability of the results through two methods: first, the average standard deviation of split frequencies was lower than 0.01 in the Bayesian method. The value of ESS was over 200. This showed that our data combined cumulatively. The results of the phylogenetic trees are presented in it [27].

## 3. Results and Discussion

### 3.1. Base Composition and Genome Organization 

**Figure 1:**
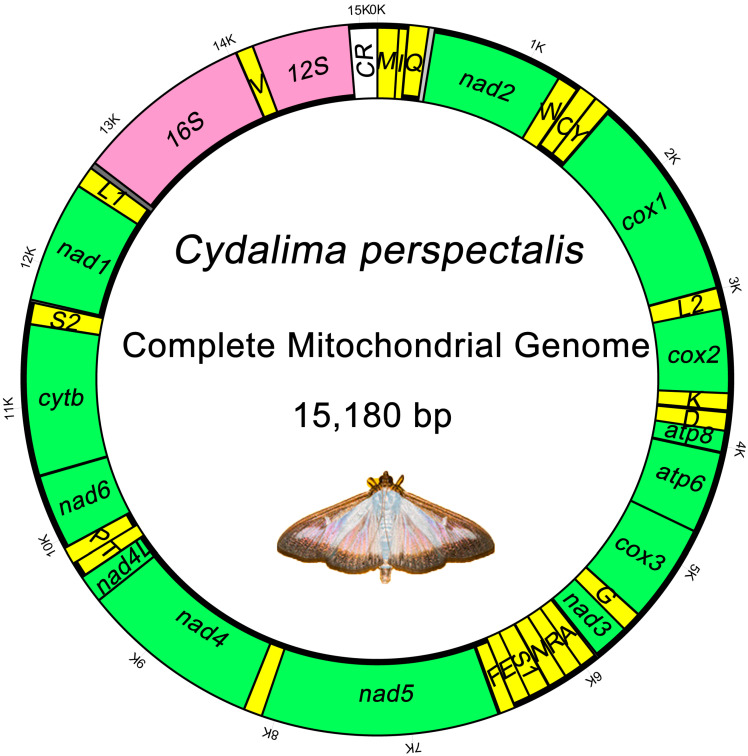
Circular map of the mitochondrial genome of *C. perspectalis*.

The complete mitogenome sequence of *C. perspectalis* is a closed circular molecule 15,180 bp in length. The composition of the gene is similar to that of other Pyraloidea insect mitogenomes such as 13 PCGs (*cox1-3*, *nad1-6*, *nad4L*, *cob*, *atp6* and *atp8*), 22 tRNA genes, two mitochondrial rRNA genes (*rrnS* and *rrnL*), and a central non-coding region known as the AT-rich region. The majority strand (F strand) encodes 23 genes. The opposite (R) strand encodes 14 genes (Figure 1, Table 2). Four of the 13 PCGs (*nad1*, *nad4*, *nad4L* and *nad5*), eight tRNAs *(trnQ*, *trnV*, *trnY*, *trnF*, *trnC*, *trnP*, *trnH,* and *trnL* [*CUN*]), and two rRNAs (*rrnS* and *rrnL*) were coded with minority-strands. The remaining 23 genes were encoded by the majority strands.

The nucleotide composition of the *C. perspetives* mitogenome is as follows (Table 3): A = 6058 (39.9%), T = 6231 (41.0%), G = 1162 (7.7%), and C = 1729 (11.4%). The A + T of the *C. perspectalis* mitogenome’s nucleotide composition was 81.0%. The entire GC-skew and AT-skew of the *C. perspectalis* mitogenome were −0.014 and −0.196, respectively. The AT skew for the *C. perspectalis* mitogenome was slightly negative. This suggests that T nucleotides are more abundant than A nucleotides. The GC-skew for the *C. perspectalis* mitogenome was scarcely negative, with C nucleotides outnumbering G nucleotides. In addition, AT-skew (0.014) and GC-skew (0.185) of the tRNAs indicate that tRNAs include more As and Gs than Ts and Cs. Similarly, AT-skew (0.050) and GC-skew (0.337) of the rRNAs clearly suggest that rRNAs have more As and Gs than Ts and Cs.

### 3.2. Protein-Coding Genes

**Figure 2:**
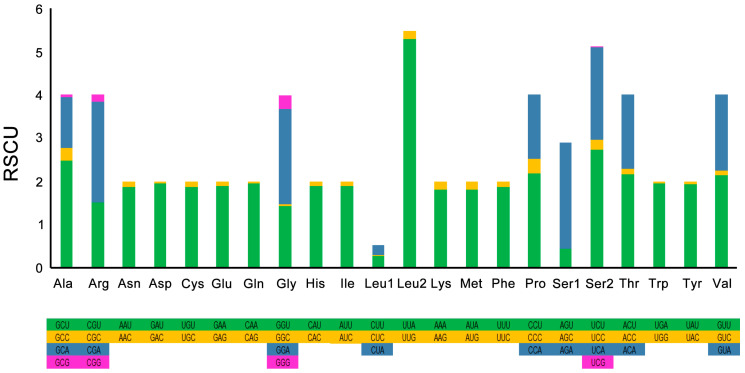
The relative synonymous codon usage (RSCU) in the mitogenome of *C. perspectalis*.

**Figure 3:**
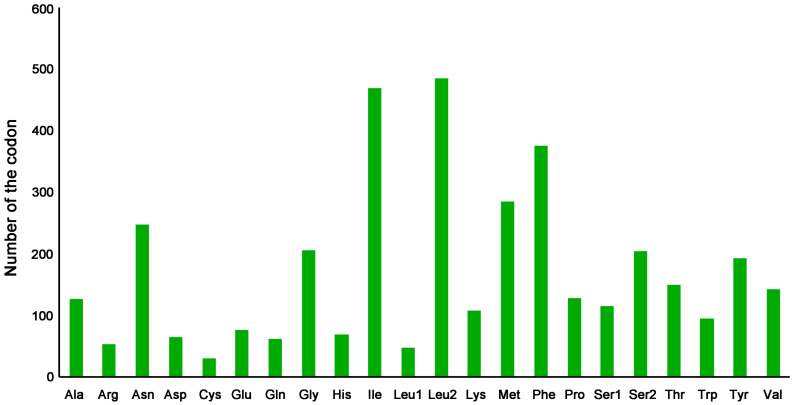
Composition of the amino acids in the mitogenome of *C. perspectalis*.

In total, 13 PCGs of *C. perspectalis* contain 3723 codons, except for the termination codons. The beginning and ending codons of 13 PCGs in the *C. perspectalis* mitogenome are presented in Table 2. The CGA codon encoded arginine, with the exception of *cox1*. All of the PCGs were launched by ATN codons. The CGA codon is incredibly protected across almost all groups of the insect [28,29,30]. In the *C. perspectalis* mitogenome, eight PCGs (*atp6*, *atp8*, *cox1*, *cox3*, *nad2*, *nad3*, *nad6,* and *cob*) had the whole stop codon TAA, but the other five ended with a single T (*nad1*, *nad4*, *nad4L*, *cox2*, and *nad5*). The ordinary A + T of the 13 PCGs was 79.6%. Moreover, 13 PCGs had a slightly negative AT skew, although it was a marginally positive GC skew (Table 3). For the *C. perspectalis* mitogenome, the related synonymous codon usage (RSCU) is valuable, as outlined in Table 4 and Figure 2, where NNT and NNA were higher than 1.0, apart from *Leu* (CUR), showing a great Ts or As bias in the 3rds. *Leu* (UUR) (484), *Ile* (469), and *Phe* (374) (Figure 3) are the most frequent amino acids found in mitochondrial proteins.

### 3.3. Control Region

The control region (AT-rich region) plays a crucial role in the introduction of the transcription and replication of the mitogenome [31]. The AT-rich part (288 bp) of the *C. perspectalis* mitogenome is situated among *trnM* and *rrnS*. The entire AT content of the PCGs was 96.2% and it was highest in the mitogenome of *C. perspectalis*. The entire GC-skew and AT-skew in the AT-rich part of *C. perspectalis* were 0.26 and 0.01, respectively (Table 3). The GC-skew and AT-skew for the AT-rich part of *C. perspectalis* were marginally positive, showing that G and A are more abundant than C and T.

**Figure 4:**
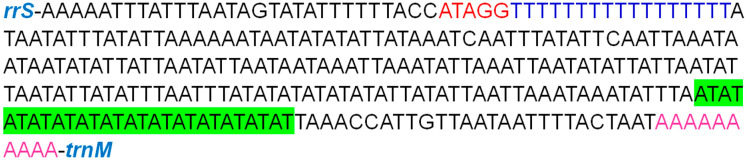
AT-rich region of the *C. perspectalis* mitogenome. Coloured nucleotides indicate the ATAGG motif (red), the poly-T stretch (blue), a microsatellite A/T repeat sequence (green), and the poly-A stretch (pink).

Some protected structures were discovered in an AT-rich part of *C. perspectalis* (Figure 4). The motif ATAGG plus 17 bp poly-T stretch downstream of *rrnS* was the first protected structure and may demonstrate the source of light strands or minority replication [32,33]. In the A + T rich region, the microsatellite-like repeat (AT)_14_ elements were detected. In addition, a 10 bp poly-A stretch was discovered just in front of the *trnM* region. Many tandem repeat elements are usually present in the A + T-rich regions of most insects. No repetitions were discovered in the A + T-rich region of the *C. perspectalis* mitogenome (Figure 4).

### 3.4. Rearrangement of Gene

**Figure 5:**
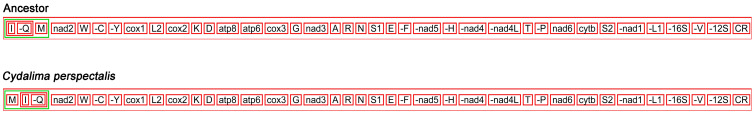
The mitochondrial gene order of *C. perspectalis* and ancestral insects. tRNA genes are indicated by singer letter IUPAC-IUB abbreviation with S1 = AGN, S2 = UCN, L1 = CUN, and L2 = UUR. Protein and rRNA genes are labelled with three letter code.

The arrangement of genes of Pyraloidea insects is often remarkably conserved. In contrast to the putative ancestral arthropod mitogenome, the order of the *C. perspectalis* differs from that of traditional insects. The *trnM* gene’s placement in the *C. perspectalis* mitogenome is *trnM*-*trnI*-*trnQ*-*nad2*. This differs from conventional insects, in which *trnM* is situated between *nad2* and *trnQ* (Figure 5). The ancestral insect placement of the *trnM* gene clusters has been discovered in ghost moths [34]. The rearrangement of genes in *C. perspectalis* stands for the opinion that the ancestral arrangement of the *trnM* gene cluster goes through rearrangement after Hepialoidea departs from the Pyraloidea lineages. Rearrangements of tRNA are believed to be the result of a tandem copy of the mitogenome’s part as a whole. This was followed by non-random or random loss of identical copies [35,36,37,38].

### 3.5. Phylogenetic Analyses

**Figure 6:**
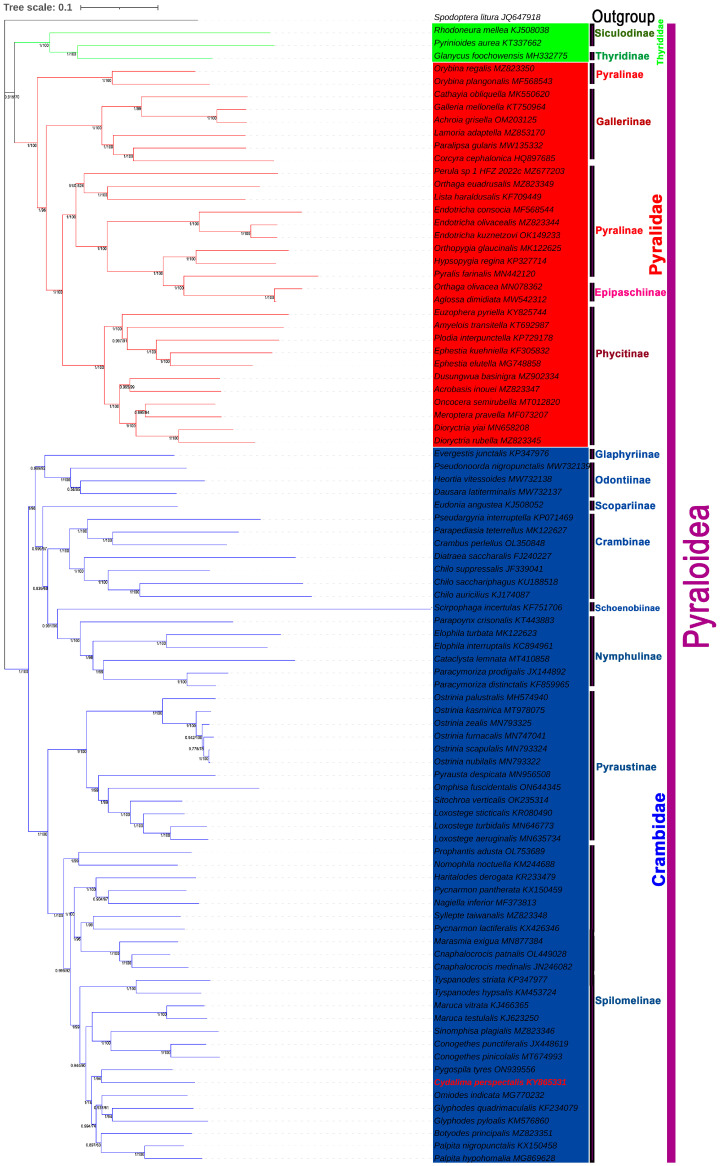
Phylogenetic tree derived for Pyraloidea using Bayesian inference (BI) and maximum likelihood (ML) analyses based on nucleotide (NT). Bootstrap value (BP) and Bayesian posterior probability (BPP) of each node are shown such as BPP based on the NT dataset/BP based on the NT dataset, 1.00/100.

Based on nucleotide alignments (NT dataset), phylogenetic trees were constructed using two methods (ML and BI) and the MAFFT alignment technique. As an outgroup, *S. litura* was used. The monophyly of every superfamily is usually strongly suggested by Bayesian inference (BI), and the maximum likelihood method based on the nucleotide sequence of 13 mitochondrial PCGs. The BI and ML trees had identical tree topologies; monophyly of the families and subfamilies was powerfully recommended, as shown by the morphological characteristics and phylogeny of the completed mitogenome [39]. In the research, the trees’ comparative analyses show high node support values, together with 13 PCG datasets (Figure 6). The phylogenetic analysis shows that *C. perspectalis* is more closely related to *Pygospila tyres* than other species, indicating that *C. perspectalis* belongs to the Spilomelinae, Crambidae, and Pyraloidea. As shown in Figure 6, the monophyly of each superfamily is generally well-supported, typically with posterior probabilities greater than 0.9 and bootstrap support (BS) greater than 75. It is obvious that three families belong to the Pyraloidea: Thyrididae, Pyralidae, and Crambidae. Regier et al. presented molecular phylogenetic research on Pyraloidea using five nuclear genes. The findings led to a new classification of Crambidae into ‘non-PS Clade’ and ‘PS Clade’. The two sister lineages correspond suitably to the ‘PS clade’ (Pyraustinae and Spilomelinae) and the ‘non-PS clade’ (Glaphyriinae, Acentropinae, Crambinae, Schoenobiinae, and Scopariinae) [40]. Our phylogenetic analysis outcome demonstrates that the same topological structures were derived from some traditional classifications and molecular data. Four of the subfamilies, Galleriinae + (Phycitinae + (Pyralinae + Epipaschiinae)) have been widely supported based on a variety of combinations of mitogenomic data or multiple gene markers in Pyralidae [40,41,42,43], and these phylogenetic relationships were also obtained based on 14 nuclear gene data. Meanwhile, the limited availability of a mitogenome precluded the Chrysauginae from being sampled in this case [44]. *Orybina* was regarded as a member of Pyralina based on the morphological method. However, a molecular phylogenetic analysis of *Orybina* revealed that the phylogenetic position was away from the Pyralina and close to Galleriinae, which is consistent with a previous study with significant value support [45]. Within the Crambidae, the ‘PS clade’ Pyraustinae and Spilomelinae formed sister lineages, while the ‘non-PS clade’ was divided into two sister lineages: one group included Glaphyriinae and Odontiinae, while the other group included the remaining four subfamilies (Schoenobiinae, Crambinae, Scopariinae, and Nymphulinae). The family-level topology of the phylogenetic analyses can be described as follows: (Glaphyriinae + Odontiinae) (Schoenobiinae + (Crambinae + (Scopariinae + Nymphulinae))) and the results were strongly supported (BS ≥ 95, PP = 1.00) and consistent with the previous research results [1,46]. Nevertheless, since we identified a separate sample in the research, a more desirable realization of the Pyraloidea mitogenome requires an extension of the genome and taxon samplings, especially in the *Orybina* and Chrysauginae.

## 4. Conclusions

In this study, we reported a complete mitogenome of *Cydalima perspectalis*, and the phylogenetic analyses of *C. perspectalis* were inferred using nucleotide sequence. The arrangement of a gene in the *C. perspectalis* mitogenome is similar to that of the Pyraloidea mitogenome. All of the PCGs were initiated by ATN codons, except for *cox1*, which was undertaken by CGA. Five genes had incomplete stop codons that contain only ‘T’. All tRNA genes displayed a typical cloverleaf structure of mitochondrial tRNA, except for *trnS1* (AGN). The control region contained an ‘ATAGG(A)’-like motif followed by a poly-T stretch. Phylogenetic analysis within Pyraloidea was constructed using the BI and ML methods. The results showed that *C. perspectalis* is more closely related to *Pygospila tyres* within Spilomelinae than those of Crambidae and Pyraloidea. These molecular-based phylogenies support the morphological classification of the relationships within the Pyraloidea species.

## Figures and Tables

**Table 1:** Pyraloidea species used in the phylogenetic analyses.

Organism	Superfamily	Family	Subfamily	Genus	ID	Length	AT%
*Chilo auricilius*	Pyraloidea	Crambidae	Crambinae	Chilo	KJ174087.1	15,367	82.1
*Chilo sacchariphagus*	Pyraloidea	Crambidae	Crambinae	Chilo	KU188518.1	15,378	81.0
*Chilo suppressalis*	Pyraloidea	Crambidae	Crambinae	Chilo	JF339041.1	15,395	80.6
*Crambus perlellus*	Pyraloidea	Crambidae	Crambinae	Crambus	OL350848.1	15,440	81.3
*Diatraea saccharalis*	Pyraloidea	Crambidae	Crambinae	Diatraea	FJ240227.1	15,490	80.1
*Parapediasia teterrellus*	Pyraloidea	Crambidae	Crambinae	Parapediasia	MK122627.1	15,368	80.5
*Pseudargyria interruptella*	Pyraloidea	Crambidae	Crambinae	Pseudargyria	KP071469.1	15,231	79.4
*Lista haraldusalis*	Pyraloidea	Pyralidae	Epipaschiinae	Lista	KF709449.1	15,213	81.5
*Orthaga euadrusalis*	Pyraloidea	Pyralidae	Epipaschiinae	Orthaga	MZ823349.1	15,268	80.2
*Orthaga olivacea*	Pyraloidea	Pyralidae	Epipaschiinae	Orthaga	MN078362.1	15,174	79.0
*Achroia grisella*	Pyraloidea	Pyralidae	Galleriinae	Achroia	OM203125.1	15,368	80.2
*Cathayia obliquella*	Pyraloidea	Pyralidae	Galleriinae	Cathayia	MK550620.1	15,408	80.6
*Corcyra cephalonica*	Pyraloidea	Pyralidae	Galleriinae	Corcyra	HQ897685.1	15,273	80.5
*Galleria mellonella*	Pyraloidea	Pyralidae	Galleriinae	Galleria	KT750964.1	15,320	80.4
*Lamoria adaptella*	Pyraloidea	Pyralidae	Galleriinae	Lamoria	MZ853170.1	15,439	80.1
*Paralipsa gularis*	Pyraloidea	Pyralidae	Galleriinae	Paralipsa	MW135332.1	15,280	79.5
*Dausara latiterminalis*	Pyraloidea	Crambidae	Odontiinae	Dausara	MW732137.1	15,147	80.5
*Eudonia angustea*	Pyraloidea	Crambidae	Scopariinae	Eudonia	KJ508052.1	15,386	81.4
*Evergestis junctalis*	Pyraloidea	Crambidae	Glaphyriinae	Evergestis	KP347976.1	15,438	81.0
*Hellula undalis*	Pyraloidea	Crambidae	Glaphyriinae	Hellula	KJ636057.1	14,678	79.9
*Heortia vitessoides*	Pyraloidea	Crambidae	Odontiinae	Heortia	MW732138.1	15,237	80.6
*Pseudonoorda nigropunctalis*	Pyraloidea	Crambidae	Odontiinae	Pseudonoorda	MW732139.1	15,084	81.0
*Rhodoneura mellea*	Pyraloidea	Thyrididae	Siculodinae	Rhodoneura	KJ508038.1	15,615	80.7
*Cataclysta lemnata*	Pyraloidea	Crambidae	Nymphulinae	Cataclysta	MT410858.1	15,333	79.5
*Elophila interruptalis*	Pyraloidea	Crambidae	Nymphulinae	Elophila	KC894961.1	15,351	80.3
*Elophila turbata*	Pyraloidea	Crambidae	Nymphulinae	Elophila	MK122623.1	15,348	81.2
*Paracymoriza distinctalis*	Pyraloidea	Crambidae	Nymphulinae	Paracymoriza	KF859965.1	15,354	82.2
*Paracymoriza prodigalis*	Pyraloidea	Crambidae	Nymphulinae	Paracymoriza	JX144892.1	15,326	81.5
*Parapoynx crisonalis*	Pyraloidea	Crambidae	Nymphulinae	Parapoynx	KT443883.1	15,374	82.0
*Acrobasis inouei*	Pyraloidea	Pyralidae	Phycitinae	Acrobasis	MZ823347.1	15,239	80.3
*Amyelois transitella*	Pyraloidea	Pyralidae	Phycitinae	Amyelois	KT692987.1	15,205	79.6
*Dioryctria rubella*	Pyraloidea	Pyralidae	Phycitinae	Dioryctria	MZ823345.1	15,422	79.8
*Dioryctria yiai*	Pyraloidea	Pyralidae	Phycitinae	Dioryctria	MN658208.1	15,430	81.0
*Dusungwua basinigra*	Pyraloidea	Pyralidae	Phycitinae	Dusungwua	MZ902334.1	15,328	80.0
*Ephestia elutella*	Pyraloidea	Pyralidae	Phycitinae	Ephestia	MG748858.1	15,346	80.7
*Ephestia kuehniella*	Pyraloidea	Pyralidae	Phycitinae	Ephestia	KF305832.2	15,327	79.8
*Euzophera pyriella*	Pyraloidea	Pyralidae	Phycitinae	Euzophera	KY825744.1	15,184	79.8
*Meroptera pravella*	Pyraloidea	Pyralidae	Phycitinae	Meroptera	MF073207.1	15,260	80.5
*Oncocera semirubella*	Pyraloidea	Pyralidae	Phycitinae	Oncocera	MT012820.1	15,290	81.4
*Plodia interpunctella*	Pyraloidea	Pyralidae	Phycitinae	Plodia	KP729178.1	15,287	80.1
*Aglossa dimidiata*	Pyraloidea	Pyralidae	Pyralinae	Aglossa	MW542312.1	15,225	79.1
*Endotricha consocia*	Pyraloidea	Pyralidae	Pyralinae	Endotricha	MF568544.1	15,201	79.7
*Endotricha kuznetzovi*	Pyraloidea	Pyralidae	Pyralinae	Endotricha	OK149233.1	15,244	80.7
*Endotricha olivacealis*	Pyraloidea	Pyralidae	Pyralinae	Endotricha	MZ823344.1	15,239	80.6
*Hypsopygia regina*	Pyraloidea	Pyralidae	Pyralinae	Hypsopygia	KP327714.1	15,212	78.7
*Orthopygia glaucinalis*	Pyraloidea	Pyralidae	Pyralinae	Orthopygia	MK122625.1	15,198	78.0
*Orybina plangonalis*	Pyraloidea	Pyralidae	Pyralinae	Orybina	MF568543.1	14,823	80.7
*Orybina regalis*	Pyraloidea	Pyralidae	Pyralinae	Orybina	MZ823350.1	15,403	81.0
*Perula* sp.	Pyraloidea	Pyralidae	Pyralinae	Perula	MZ677203.1	15,252	81.0
*Pyralis farinalis*	Pyraloidea	Pyralidae	Pyralinae	Pyralis	MN442120.1	15,204	78.1
*Cnaphalocrocis medinalis*	Pyraloidea	Crambidae	Pyraustinae	Cnaphalocrocis	JN246082.1	15,388	82.0
*Cnaphalocrocis patnalis*	Pyraloidea	Crambidae	Pyraustinae	Cnaphalocrocis	OL449028.1	15,305	81.8
*Loxostege aeruginalis*	Pyraloidea	Crambidae	Pyraustinae	Loxostege	MN635734.1	15,339	80.1
*Loxostege sticticalis*	Pyraloidea	Crambidae	Pyraustinae	Loxostege	KR080490.1	15,218	80.8
*Loxostege turbidalis*	Pyraloidea	Crambidae	Pyraustinae	Loxostege	MN646773.1	15,240	80.0
*Marasmia exigua*	Pyraloidea	Crambidae	Pyraustinae	Marasmia	MN877384.1	15,262	81.6
*Ostrinia furnacalis*	Pyraloidea	Crambidae	Pyraustinae	Ostrinia	MN747041.1	15,241	80.9
*Ostrinia kasmirica*	Pyraloidea	Crambidae	Pyraustinae	Ostrinia	MT978075.1	15,214	81.0
*Ostrinia nubilalis*	Pyraloidea	Crambidae	Pyraustinae	Ostrinia	MN793322.1	15,248	80.9
*Ostrinia palustralis*	Pyraloidea	Crambidae	Pyraustinae	Ostrinia	MH574940.1	15,246	80.6
*Ostrinia scapulalis*	Pyraloidea	Crambidae	Pyraustinae	Ostrinia	MN793324.1	15,311	81.0
*Ostrinia zealis*	Pyraloidea	Crambidae	Pyraustinae	Ostrinia	MN793325.1	15,208	80.9
*Pyrausta despicata*	Pyraloidea	Crambidae	Pyraustinae	Pyrausta	MN956508.1	15,389	80.9
*Sitochroa verticalis*	Pyraloidea	Crambidae	Pyraustinae	Sitochroa	OK235314.1	15,275	80.6
*Syllepte taiwanalis*	Pyraloidea	Crambidae	Pyraustinae	Syllepte	MZ823348.1	15,264	81.7
*Scirpophaga incertulas*	Pyraloidea	Crambidae	Schoenobiinae	Scirpophaga	KF751706.1	15,220	77.2
*Pyrinioides aurea*	Pyraloidea	Thyrididae	Siculodinae	Pyrinioides	KT337662.1	15,362	80.0
*Botyodes principalis*	Pyraloidea	Crambidae	Spilomelinae	Botyodes	MZ823351.1	15,262	80.7
*Conogethes pinicolalis*	Pyraloidea	Crambidae	Spilomelinae	Conogethes	MT674993.1	15,336	80.1
*Conogethes punctiferalis*	Pyraloidea	Crambidae	Spilomelinae	Conogethes	JX448619.1	15,355	80.6
*Cydalima perspectalis*	Pyraloidea	Crambidae	Spilomelinae	Cydalima	KY865331.1	15,180	80.9
*Glyphodes pyloalis*	Pyraloidea	Crambidae	Spilomelinae	Glyphodes	KM576860.1	14,960	80.7
*Glyphodes quadrimaculalis*	Pyraloidea	Crambidae	Spilomelinae	Glyphodes	KF234079.1	15,255	80.8
*Haritalodes derogata*	Pyraloidea	Crambidae	Spilomelinae	Haritalodes	KR233479.1	15,253	80.7
*Maruca testulalis*	Pyraloidea	Crambidae	Spilomelinae	Maruca	KJ623250.1	15,110	80.8
*Maruca vitrata*	Pyraloidea	Crambidae	Spilomelinae	Maruca	KJ466365.1	15,385	80.7
*Nagiella inferior*	Pyraloidea	Crambidae	Spilomelinae	Nagiella	MF373813.1	15,348	81.5
*Nomophila noctuella*	Pyraloidea	Crambidae	Spilomelinae	Nomophila	KM244688.1	15,309	81.4
*Omiodes indicata*	Pyraloidea	Crambidae	Spilomelinae	Omiodes	MG770232.1	15,367	81.6
*Omphisa fuscidentalis*	Pyraloidea	Crambidae	Spilomelinae	Omphisa	ON644345.1	15,347	79.0
*Palpita hypohomalia*	Pyraloidea	Crambidae	Spilomelinae	Palpita	MG869628.1	15,271	81.0
*Palpita nigropunctalis*	Pyraloidea	Crambidae	Spilomelinae	Palpita	KX150458.1	15,226	81.0
*Prophantis adusta*	Pyraloidea	Crambidae	Spilomelinae	Prophantis	OL753689.1	15,689	81.5
*Pycnarmon lactiferalis*	Pyraloidea	Crambidae	Spilomelinae	Pycnarmon	KX426346.1	15,219	81.7
*Pycnarmon pantherata*	Pyraloidea	Crambidae	Spilomelinae	Pycnarmon	KX150459.1	15,545	81.4
*Pygospila tyres*	Pyraloidea	Crambidae	Spilomelinae	Pygospila	ON939556.1	15,287	81.3
*Sinomphisa plagialis*	Pyraloidea	Crambidae	Spilomelinae	Sinomphisa	MZ823346.1	15,214	80.6
*Spoladea recurvalis*	Pyraloidea	Crambidae	Spilomelinae	Spoladea	KJ739310.1	15,273	80.9
*Tyspanodes hypsalis*	Pyraloidea	Crambidae	Spilomelinae	Tyspanodes	KM453724.1	15,329	81.4
*Tyspanodes striata*	Pyraloidea	Crambidae	Spilomelinae	Tyspanodes	KP347977.1	15,255	81.3
*Glanycus foochowensis*	Pyraloidea	Thyrididae	Thyridinae	Glanycus	MH332775.1	15,430	81.3

**Table 2:** Summary of the mitogenome of *C. perspectalis*.

Gene	Strand	Location	Size (bp)	Intergenic Length	Anticodon	Start Codon	Stop Codon
trnM	F	1–68	68	0	CAT	–	–
trnI	F	69–134	66	−3	GAT	–	–
trnQ	R	132–200	69	43	TTG	–	–
nad2	F	244–1257	1014	1	–	ATT	TAA
trnW	F	1259–1325	67	−8	TCA	–	–
trnC	R	1318–1383	66	3	GCA	–	–
trnY	R	1387–1451	65	9	GTA	–	–
cox1	F	1461–2996	1536	−5	–	CGA	TAA
trnL2	F	2992–3058	67	0	TAA	–	–
cox2	F	3059–3740	682	0	–	ATG	T
trnK	F	3741–3811	71	13	CTT	–	–
trnD	F	3825–3890	66	0	GTC	–	–
atp8	F	3891–4052	162	−7	–	ATC	TAA
atp6	F	4046–4720	675	−1	–	ATG	TAA
cox3	F	4720–5508	789	2	–	ATG	TAA
trnG	F	5511–5576	66	0	TCC	–	–
nad3	F	5577–5930	354	16	–	ATT	TAA
trnA	F	5947–6011	65	0	TGC	–	–
trnR	F	6012–6074	63	1	TCG	–	–
trnN	F	6076–6141	66	6	GUU	–	–
trnS1	F	6148–6213	66	0	GCT	–	–
trnE	F	6214–6280	67	2	TTC	–	–
trnF	R	6283–6349	67	12	GAA	–	–
nad5	R	6362–8098	1737	0	–	ATA	T
trnH	R	8099–8164	66	1	GTG	–	–
nad4	R	8166–9506	1341	1	–	ATC	T
nad4L	R	9508–9798	291	2	–	ATA	T
trnT	F	9801–9867	67	0	TGT	–	–
trnP	R	9868–9933	66	2	TGG	–	–
nad6	F	9936–10,472	537	6	–	ATT	TAA
cob	F	10,479–11,630	1152	−2	–	ATG	TAA
trnS2	F	11,629–11,693	65	17	TGA	–	–
nad1	R	11,711–12,649	939	1	–	ATA	T
trnL1	R	12,651–12,724	74	−44	TAG	–	–
16S	R	12,681–14,050	1370	4	–	–	–
trnV	R	14,055–14,124	70	−1	TAC	–	–
12S	R	14,124–14,892	769	0	–	–	–
A + T-rich		14,893–15,180	288	0	–	–	–

**Table 3:** Composition and skewness in the *C. perspectalis* mitogenome.

Regions	Size (bp)	T	C	A	G	AT (%)	GC (%)	AT Skewness	GC Skewness
Full genome	15,180	41	11.4	39.9	7.7	80.9	19.1	−0.014	−0.196
PCGs	11,202	45.5	9.7	34.1	10.7	79.6	20.4	−0.144	0.046
tRNAs	1473	40.3	7.5	41.4	10.9	81.7	18.4	0.014	0.185
rRNAs	2139	40.1	5.1	44.4	10.4	84.5	15.5	0.05	0.337
Control region	288	48.6	2.4	47.6	1.4	96.2	3.8	0.01	0.26

**Table 4:** Codon number and RSCU in the *C. perspectalis* mitochondrial PCGs. (* the termination codons).

Codon	Count	RSCU	Codon	Count	RSCU	Codon	Count	RSCU	Codon	Count	RSCU
UUU(F)	350	1.87	UCU(S)	109	2.73	UAU(Y)	185	1.92	UGU(C)	28	1.87
UUC(F)	24	0.13	UCC(S)	9	0.23	UAC(Y)	8	0.08	UGC(C)	2	0.13
UUA(L)	467	5.28	UCA(S)	85	2.13	UAA(*)	11	2	UGA(W)	91	1.94
UUG(L)	17	0.19	UCG(S)	1	0.03	UAG(*)	0	0	UGG(W)	3	0.06
CUU(L)	23	0.26	CCU(P)	69	2.17	CAU(H)	65	1.88	CGU(R)	20	1.51
CUC(L)	2	0.02	CCC(P)	11	0.35	CAC(H)	4	0.12	CGC(R)	0	0
CUA(L)	22	0.25	CCA(P)	47	1.48	CAA(Q)	60	1.94	CGA(R)	31	2.34
CUG(L)	0	0	CCG(P)	0	0	CAG(Q)	2	0.06	CGG(R)	2	0.15
AUU(I)	442	1.88	ACU(T)	80	2.15	AAU(N)	231	1.87	AGU(S)	17	0.43
AUC(I)	27	0.12	ACC(T)	5	0.13	AAC(N)	16	0.13	AGC(S)	0	0
AUA(M)	258	1.81	ACA(T)	64	1.72	AAA(K)	97	1.8	AGA(S)	98	2.46
AUG(M)	27	0.19	ACG(T)	0	0	AAG(K)	11	0.2	AGG(S)	0	0
GUU(V)	76	2.14	GCU(A)	78	2.48	GAU(D)	62	1.94	GGU(G)	73	1.42
GUC(V)	4	0.11	GCC(A)	9	0.29	GAC(D)	2	0.06	GGC(G)	2	0.04
GUA(V)	62	1.75	GCA(A)	37	1.17	GAA(E)	72	1.89	GGA(G)	114	2.22
GUG(V)	0	0	GCG(A)	2	0.06	GAG(E)	4	0.11	GGG(G)	16	0.31

## Data Availability

The datasets generated for this study can be found in the GenBank accession no. KY865331.
